# Has the cesarean epidemic in Czechia been reversed despite fertility postponement?

**DOI:** 10.1186/s12884-022-04781-1

**Published:** 2022-06-06

**Authors:** Tomáš Fait, Anna Šťastná, Jiřina Kocourková, Eva Waldaufová, Luděk Šídlo, Michal Kníže

**Affiliations:** 1 Department of Gynecology and Obstetrics, Second Faculty of Medicine, Charles University and Motol University Hospital, Prague, Czechia; 2 Department of Demography and Geodemography, Faculty of Science, Charles University, Prague, Czechia

**Keywords:** Cesarean section (CS), Fertility, Fertility postponement, Czechia, Health status, Breech delivery

## Abstract

**Background:**

Although the percentage of cesarean sections (CS) in Czechia is below the average of that of other developed countries (23.6%), it still exceeds WHO recommendations (15%). The first aim of the study is to examine the association between a CS birth and the main health factors and sociodemographic characteristics involved, while the second aim is to examine recent trends in the CS rate in Czechia.

**Methods:**

Anonymized data on all mothers in Czechia for 2018 taken from the National Register of Expectant Mothers was employed. The risk of cesarean delivery for the observed factors was tested via the construction of a binary logistic regression model that allowed for adjustments for all the other covariates in the model.

**Results:**

Despite all the covariates being found to be statistically significant, it was determined that health factors represented a higher risk of a CS than sociodemographic characteristics. A previous CS was found to increase the risk of its recurrence by 33 times (OR = 32.96, 95% CI 30.95–35.11, *p*<0.001). The breech position increased the risk of CS by 31 times (OR = 31.03, 95% CI 28.14–34.29, *p*<0.001). A multiple pregnancy increased the odds of CS six-fold and the use of ART 1.8-fold. Mothers who suffered from diabetes before pregnancy were found to be twice as likely to give birth via CS (OR = 2.14, 95% CI 1.76–2.60, *p*<0.001), while mothers with gestational diabetes had just 23% higher odds of a CS birth (OR = 1.23, 95% CI 1.16–1.31, *p*<0.001). Mothers who suffered from hypertension gave birth via CS twice as often as did mothers without such complications (OR = 2.01, 95% CI 1.86–2.21, *p*<0.001).

**Conclusions:**

The increasing age of mothers, a significant risk factor for a CS, was found to be independent of other health factors. Accordingly, delayed childbearing is thought to be associated with the increase in the CS rate in Czechia. However, since other factors come into play, further research is needed to assess whether the recent slight decline in the CS rate is not merely a temporal trend.

## Introduction

**Fig. 1:**
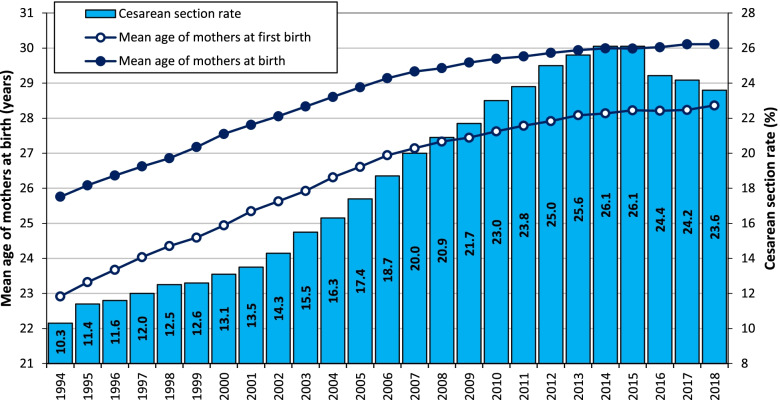
Mean age of mothers at birth, first births and the CS rate, Czechia, 1994–2018. Source: [6–11]

**Fig. 2:**
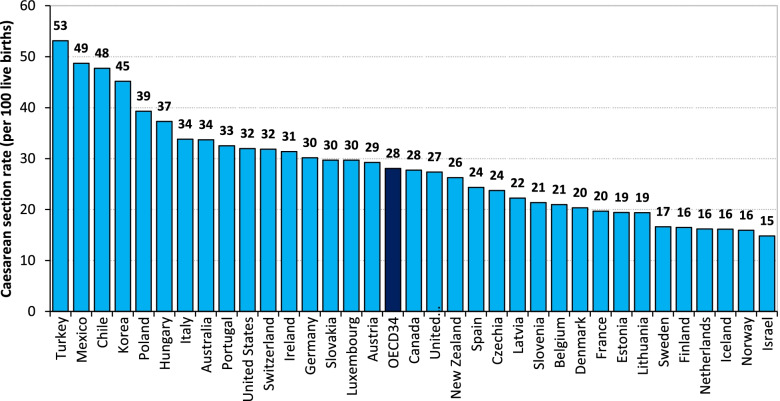
Cesarean section rate in OECD countries in 2017. Source: [4]

Cesarean section (CS), when used appropriately, should account for 10–15% of births [1, 2]. In recent years, however, the trend toward the use of CS in obstetric practice has been on the increase worldwide. Eastern Europe witnessed one of the highest increases (two-fold) in the use of CS in the period 2000–2015 [3, 4]. This trend was particularly marked in Czechia, where the CS rate increased from 10.3% in 1994 to a maximum value of 26.1% in 2015, followed by a slight decrease to 23.6% in 2018 (Fig. 1). Currently, the CS rate in Czechia is below the average of other developed countries [3–5] (Fig. 2).

One of the most likely reasons for this phenomenon concerns the dynamic increase in the age of mothers [12], which represents a significant recent demographic trend in Czechia [13–15]. Between 1994 and 2015 the mean age of mothers increased on a continuous basis, as did the share of CS, both of which stagnated only recently (Fig. 1). Fertility postponement is further connected with a decrease in the probability of having a second child [16, 17], the increased use of assisted reproduction methods [18, 19], and health risks for both mothers and their children [20, 21], i.e. factors which are also related to the increased use of CS [22, 23].

The reasons for the increase in the CS are multifactorial and include health care practices [2, 3]. The care of pregnant women in Czechia is fully entrusted to gynecologists and obstetricians. It is strongly recommended that the birth should take place in a medical facility and, even if it is conducted by a midwife, the doctor remains the legally responsible person. The decision on a planned CS cannot be based on a request from the mother. While some maternity facilities are run by private companies, all the health care facilities used by Czech citizens are covered by the public health insurance system under the same conditions.

The first aim of the article is to evaluate, taking Czechia as an example, the association between the use of CS and the main medical factors related to the increased use of CS (complications during pregnancy and childbirth, diabetes, gestational age, the birth weight, the breech position, repeat CS, singleton/multiple pregnancy, and conception method) and to subsequently compare these associations with those between the use of CS and sociodemographic characteristics (the age of the mother, the birth order, marital status and the mother’s level of education). The second aim is to examine recent trends in the CS rate in Czechia.

## Data and methodology

The study employed a unique data source that contains anonymized data on all mothers in Czechia for 2018 obtained from the National Registry of Mothers at Childbirth (NRMC), which is managed by the Institute of Health Information and Statistics of the Czech Republic (IHIS CR) [7]. The data contained in the National Register is based on the so-called report on the mother at childbirth, a mandatory statistical report that is completed on all mothers, including foreigners, who give birth in Czechia. Data on the CS rate in the private sector is not reported separately.

In 2018, a total of 111,749 mothers gave birth to 113,234 children; 6.2% of them had non-Czech citizenship [7]. Since one of the most important considerations concerning the study of cesarean births is whether ART was used to achieve pregnancy, information on the date of embryo transfer was added to the data set by linking the file from the NRMC with the respective file obtained from the National Register of Assisted Reproduction (NRAR) using the mothers’ so-called birth numbers (a unique number that is assigned to all Czechs at birth). Based on the comparison of the date of birth and the date of embryo transfer, it was possible to estimate those pregnancies that resulted from the use of ART.

Firstly, a descriptive analysis of the relationships between the observed variables was conducted so as to evaluate the distribution of the increased incidence of CS births according to the various factors considered. Most of the monitored variables contained data on all the mothers, with the exception of marital status and level of education; the completion of these questions is optional. There was a lack of information on 673 mothers concerning marital status (0.6% of the total sample) and on 23,113 mothers in the case of the level of education (20.7% of the total sample). Cesarean deliveries were divided into planned and acute.

In order to assess the association between the various covariates and the risk of CS, a binary logistic regression model was constructed, which enabled the testing of the association of the various variables on the incidence of CS births (1 yes, 0 no), assuming all the other characteristics of the mothers were equal. The application of the binary logistic regression model allowed for the removal of the mutual influence of the covariates and the testing of whether they also acted individually, all else being equal.

Two logistic regression models were constructed. Model 1 included all the mothers except for those for whom no data was available on the marital status and level of education (*N* = 88,041, i.e. 79% of the total number of mothers), while Model 2 included only those mothers who had already given birth in the past (a total of 46,127 mothers, i.e. 80% of repeat mothers after excluding women with no data for marital status and/or education).

The binary logistic regression model was used to explain the effects of the explanatory variables on the dependent variable “having a childbirth via cesarean section” (Y = 1 for cesarean section, otherwise Y = 0). *x* = *(x1, …. xk)*’ is the vector of the explanatory variables:

$$\mathrm{logit}\left({\text{Pr}}\langle Y=1|x\rangle \right)={\text{log}}\left\{\frac{{\text{Pr}}\langle Y=1|x\rangle }{1-{\text{Pr}}\langle Y=1|x\rangle }\right\}={\beta }_{0}+{x}^{\mathrm{^{\prime}}}\beta ,$$

where β_0_ is the intercept parameter and β is the vector of the slope parameters.

For the sake of clarity, the results were interpreted in terms of odds ratios (OR), which qualify the variables that indicate the odds of cesarean delivery for each category compared to the given reference category.

A number of demographic, health and socio-economic characteristics were included in the models as explanatory variables. With the exception of the age of the mother at childbirth (continuous), all the following covariates were categorical and were transformed into dummy variables:

- *Marital status* was divided into four categories: single, married (ref.), divorced and widowed.
- The highest attained *level of education* was divided into four groups: basic (including incomplete), secondary without the school leaving certificate (SLC), secondary with the SLC (ref.) and tertiary.
- The WHO classification of premature babies [24] was used for the categorization of the gestational age, namely: extremely preterm (less than 28 weeks), very preterm (28 to < 32 weeks), and moderate to late preterm (32 to < 37 weeks). The post-term birth category was defined according to a report by Spong [25], which indicates a post-term delivery as occurring from the 42nd week of pregnancy. A gestational age of 37–41 weeks was used as the reference category.
- The *birth order* was divided into 3 categories, namely women who had not yet given birth (ref.), women who had given birth for a second time, and women with third and higher order births.
- *Singleton* (ref.) *versus multiple pregnancy*.
- *Previous CS birth* (only in model 2, in which first-time mothers did not feature): no (ref.), yes.
- The *probable method of pregnancy of the women* was estimated based on the embryo transfer date reported for 4,018 mothers. This method of assisted reproduction was used by 3.6% of mothers. This variable was then divided into two categories – without the use of ART (ref.) and following ART.
- The *incidence of diabetes* in the mothers was divided into three categories: not detected (ref.), detected prior to pregnancy, detected during pregnancy.
- Hypertension and threatened preterm labor, which are among the most common health complications, were identified as *serious complications during pregnancy and childbirth*. Other complications (bleeding in the first, second and third trimesters, placenta previa, placental abruption and other placental abnormalities, cardiovascular complications, preeclampsia, intra-uterine growth restriction and others) were combined in the “other complications” category.
- The models also considered the incidence of a breech presentation. This variable was assigned the values: no (ref.) and yes. In the case of multiple pregnancies, the pregnancy was classified as “yes” if at least one of the children was in the breech position.

The *birth weight* was not included in the regression model since it is not considered to be a key indicator of CS. The preferred routine adopted by the field of obstetrics in Czechia comprises the evaluation of placental functioning applying the ultrasonographic measurement of flow through the umbilical artery and the middle cerebral artery. The birth weight is recorded only following the birth of the child; data on the estimated birth weight of the child prior to the birth does not form a part of the official data on which a decision on a CS is based.

In addition, the variable birth weight, which has a high degree of multicollinearity with the gestational age, was monitored in the descriptive analysis via the following 5 categories: extremely low birth weight (< 1,000 g), very low birth weight (1,000–1,499 g), low birth weight (1,500–2,499 g), normal birth weight (2,500–3,999 g) and high birth weight (≥ 4,000 g) [26]. The analysis considered the lowest birth weight in the case of multiple births.

The analysis was performed using SPSS Statistics 26 software. The findings and the discussion are reported according to STROBE Statement guidelines [27].

## Results

In 2018, a total of 111,749 mothers gave birth to 113,234 children in Czechia [7]. The highest proportion of mothers comprised the 30–34 age group (34.6%), followed by the 25–29 age group (30.5%).

In 2018, 6.9% of mothers gave birth prematurely in Czechia and 48.2% of all mothers gave birth to their first child. 35.3% of mothers had second-order births and the remaining 16.5% had third-order and higher births. 10.5% of mothers had experienced at least one previous CS birth. 1,464 sets of twins were born in Czechia in 2018, i.e. 1.3% of all births.

The share of CS births in 2018 was 23.6%. The highest proportion of CS births concerned elective CS planned during pregnancy (42.9%) and, together with elective CS, but performed during labour (7.8%), accounted for a total of 50.7%. Emergent cesarean sections performed during labor accounted for 33.5%, and during pregnancy 15.8%. Of all women who gave birth via CS (23,341) 62% were aged 30 and over. 31.8% of all CS births were repeat CS births, of which 18.9% were breech presentations, 6.4% followed ART and 4.4% were multiple pregnancies.

### Differences in the frequency of CS deliveries by socio-demographic characteristics

The distribution of CS according to age categories indicated an increasing risk with the age of the mother (Table 1). The lowest share of CS births referred to the up to 19 years age category (15.8%), with higher proportions in each subsequent age category. Compared to the total proportion of births via CS of 23.6%, the up to 29 years age group had a lower share than the average, and the 30–34 years category corresponded to the average. A significantly higher proportion of CS births concerned mothers aged 35–39 and over 40, for whom 37.2% of pregnancies ended in CS births. Conversely, the share of women with a vaginal delivery decreased with age from 84.2% before the age of 20 to 62.8% for women aged 40 and over.

**Table 1:** Cesarean delivery by the age of the mother at delivery, Czechia, 2018

Cesarean delivery		Age of the mother at delivery						
		≤ 19	20–24	25–29	30–34	35–39	≥ 40	Total
No	number	2,055	10,265	26,962	29,450	13,824	2,852	85,408
	share in %	84.2	80.9	79.2	76.2	71.3	62.8	76.4
Yes	number	387	2,425	7,098	9,193	5,552	1,686	26,341
	share in %	15.8	19.1	20.8	23.8	28.7	37.2	23.6
Total	number	2,442	12,690	34,060	38,643	19,376	4,538	111,749
	share in %	100.0	100.0	100.0	100.0	100.0	100.0	100.0

Source: [7], own calculations

**Fig. 3:**
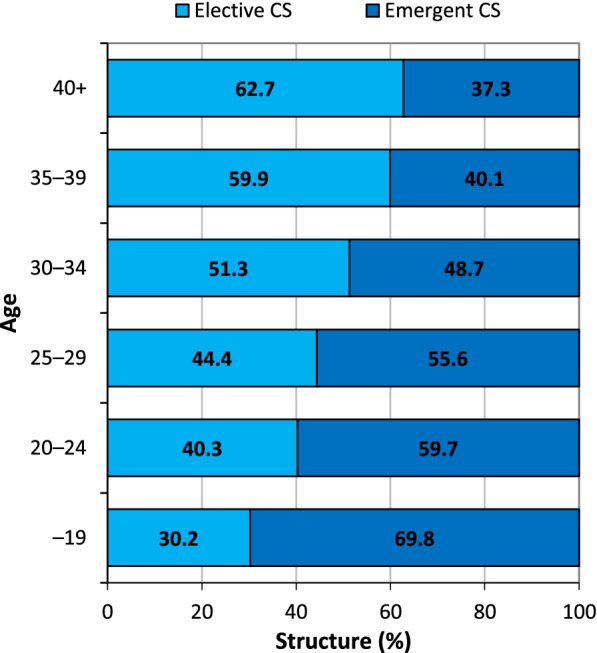
Elective and emergent CS by maternal age at delivery, Czechia, 2018. Source: [7], own calculations

In addition, the ratio of planned and emergent CS also varied depending on the age of the mother (Fig. 3), i.e. the share of planned CS births increased with age. Compared to the youngest mother age group (up to 19 years of age), concerning whom 30.2% of all CS births were planned, the over 40 years age group featured more than twice the percentage of mothers with planned CS.

**Fig. 4:**
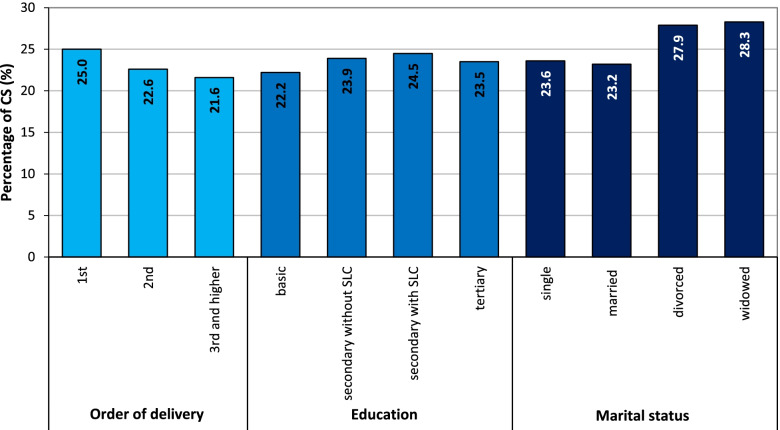
Percentage of CS of all deliveries for the given category of mothers, Czechia, 2018. Source: [7], own calculations

The proportion of CS births varied slightly depending on the birth order (Fig. 4). The highest share concerned first-time mothers (25.0%) and the lowest share mothers of third and higher birth orders (21.6%). Slight differences were also observed with respect to the education attained and the marital status of the mothers (Fig. 4). Divorced women (27.9%) and widows (28.3%) gave birth via CS more frequently than did single (23.6%) and married (23.2%) women. A lower share of CS births was observed for women with basic and incomplete education levels (22.2%), and the highest share for secondary school (with SLC) graduates (24.5%).

### Differences in the frequency of CS deliveries by health indication

Significant differences were determined with respect to the number of pregnancies and whether or not the mother had previously given birth via CS (Table 2). Multiple births were predominantly via CS (78.7%) as were births by women who had previously had a CS birth (71.2%).

**Table 2:** Percentage of CS according to a previous CS, singleton/multiple pregnancy and breech presentation, Czechia, 2018

		% of CS	Number of CS
CS for a previous delivery	no	28.8	3,386
	yes	71.2	8,372
Pregnancy	singleton	22.8	25,181
	multiple	78.7	1,160
Breech presentation	no	20.1	21,355
	yes	89.1	4,986

Source: [7], own calculations

**Fig. 5:**
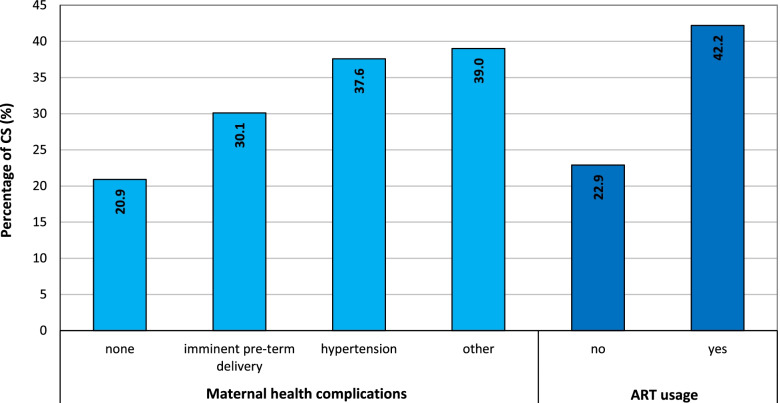
Percentage of CS according to maternal health complications and ART usage, Czechia, 2018. Source: [7], own calculations

The proportion of CS births increased in proportion to the occurrence of complications during pregnancy and childbirth (Fig. 5). The risk of CS was higher for those mothers at risk of a pre-term delivery (30.1%). Women with hypertension (37.6%) and other complications (39.0%) gave birth via CS almost twice as often as did women without health complications (20.9%).

The incidence of various types of complications during pregnancy and childbirth also varied depending on the age of the mother. Complications such as first and third trimester bleeding, placenta previa, placental abruption and other placental abnormalities, cardiovascular complications and hypertension primarily affected mothers over 35 years of age, and even more significantly mothers over 40 years of age. Conversely, some complications were characteristic of younger mothers under 24 years of age, specifically the occurrence of a significantly higher proportion of the threat of pre-term birth and intra-uterine growth restriction. The incidence of other health complications (preeclampsia, bleeding in the second trimester) did not differ significantly according to the age of the mother.

However, the proportion of diabetes, especially diabetes that was detected during pregnancy, increased with the age of the mothers (Table 3); while 6.2% of mothers aged 25–29 were affected, 11.6% of mothers aged over 40 suffered from this condition. 40% of women with preexisting diabetes gave birth via CS, while 29.9% of women with gestational diabetes and 23% of women without diabetes had CS births.

**Table 3:** Diabetes by the age of the mother at delivery, Czechia, 2018

Diabetes		Age of the mother at delivery						
		≤ 19	20–24	25–29	30–34	35–39	≥ 40	Total
No/not detected	number	2,339	11,929	31,743	35,504	17,437	3,972	102,924
	share in %	95.8	94.0	93.2	91.9	90.0	87.5	92.1
Before pregnancy	number	11	63	193	246	161	41	715
	share in %	0.5	0.5	0.6	0.6	0.8	0.9	0.6
During pregnancy	number	92	698	2124	2,893	1,778	525	8,110
	share in %	3.8	5.5	6.2	7.5	9.2	11.6	7.3
Total	number	2,442	12,690	34,060	38,643	19,376	4,538	111,749
	share in %	100.0	100.0	100.0	100.0	100.0	100.0	100.0

Source: [7], own calculations

CS delivery was observed to be less frequent for women who became pregnant without ART (22.9%) than those who underwent assisted reproduction techniques (42.2%) (Fig. 5).

Of all children born via CS, 18.6% were in the breech presentation, 3% in the transverse and oblique lie and 78.4% were in the vertex presentation. Only 9.8% of children in the breech presentation were born spontaneously.

**Fig. 6:**
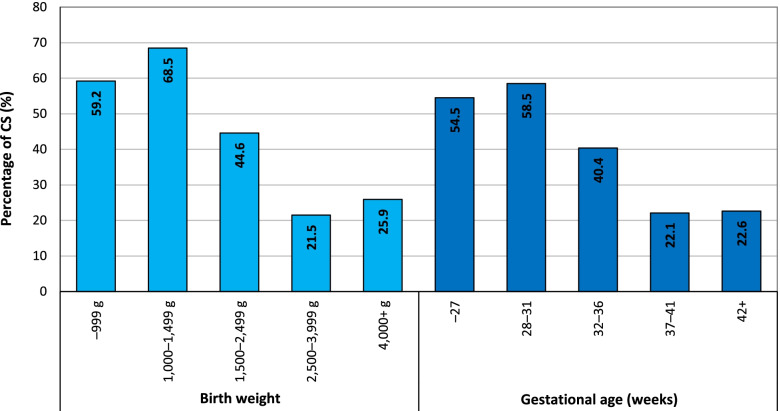
Percentage of CS of all deliveries according to birth weight and gestational age, Czechia, 2018. Source: [7], own calculations

The proportion of CS births varied significantly according to the birth weight of the child (Fig. 6). CS was significantly more common in the case of newborns who weighed less than 2,500 g than for those with normal birth weights. The highest share of CS births concerned the very low weight category (68.5%). A higher proportion of CS births was also recorded for children with higher birth weights (25.9%) than for those with normal birth weights (21.5%).

The final observed change concerned the gestational age. Significant differences were observed between mothers of gestational ages ranging from 22 to 45 weeks. The proportion of CS was significantly higher for pre-term births than for term births, while the share was slightly higher for post-term births (Fig. 6). Extremely pre-term and very pre-term births took place via CS in more than half of all such cases, while moderate to late pre-term births involved CS in 40.4% of such cases. The incidence of pre-term births was higher for women younger than 25 years and older than 40 years than for those aged 20–34.

### Health factors versus the socio-demographics associated with the increased odds of a CS delivery

The sociodemographic characteristics and health status of all the women were analyzed together employing binary logistic regression in order to identify the covariates associated with the increased odds of a CS delivery. All the covariates were entered into Model 1 for 88,041 mothers (i.e. 79% of all mothers).

The results revealed that the odds of a cesarean birth increases with the maternal age (Table 4 – Model 1). Thus, the increasing age of mothers is an important covariate associated with the increasing incidence of CS births, even when it is adjusted for relevant confounders—other age-dependent risk characteristics (e.g. pregnancy complications, the use of ART and multiple births).

**Table 4:** Odds Ratios (Exp(B)) of undergoing a cesarean delivery, Czechia, 2018

	**Model 1**					**Model 2**				
	B	Exp(B)	*p*-value	95% C.I. For Exp(B)		B	Exp(B)	*p*-value	95% C.I. For Exp(B)	
**Age of mother (cont.)**	0.055	1.06	0.000	1.053	1.061	0.043	1.04	0.000	1.036	1.051
**Gestational Age (weeks)**										
–27	0.560	1.75	0.000	1.353	2.265	0.625	1.87	0.007	1.190	2.932
28–31	1.079	2.94	0.000	2.332	3.710	1.268	3.56	0.000	2.321	5.446
32–36	0.504	1.66	0.000	1.541	1.778	0.772	2.16	0.000	1.902	2.461
37–41		1					1			
42 and more	0.251	1.29	0.000	1.142	1.447	-0.165	0.85	0.224	0.649	1.106
**Order of delivery**										
1st		1				x	x	x	x	x
2nd	-0.111	0.89	0.000	0.861	0.930		1			
3dr and higher	-0.285	0.75	0.000	0.713	0.793	-0.360	0.70	0.000	0.651	0.748
**CS in previous delivery (model 2 only)**										
No							1			
Yes						3.495	32.96	0.000	30.948	35.106
**Pregnancy**										
Singleton		1					1			
Multiple	1.805	6.08	0.000	5.132	7.211	2.191	8.94	0.000	6.928	11.537
**ART usage**										
No		1					1			
Yes	0.604	1.83	0.000	1.685	1.986	0.730	2.08	0.000	1.746	2.467
**Diabetes**										
No / not detected		1					1			
Before pregnancy	0.762	2.14	0.000	1.763	2.604	1.056	2.88	0.000	2.083	3.971
During pregnancy	0.206	1.23	0.000	1.155	1.307	0.209	1.23	0.000	1.112	1.367
**Complications in pregnancy and childbirth**										
None		1					1			
Imminent preterm delivery	-0.270	0.763	0.000	0.664	0.877	-0.363	0.70	0.003	0.547	0.885
Hypertenses	0.697	2.007	0.000	1.825	2.209	0.702	2.02	0.000	1.680	2.424
Other	0.708	2.029	0.000	1.932	2.131	0.626	1.87	0.000	1.717	2.039
**At least one child in the breech position**										
No		1					1			
Yes	3.436	31.06	0.000	28.139	34.291	3.574	35.64	0.000	30.617	41.499
**Education**										
Basic	0.044	1.04	0.198	0.977	1.117	-0.096	0.91	0.091	0.812	1.015
Secondary without SLC	-0.005	1.00	0.850	0.950	1.044	-0.049	0.95	0.244	0.876	1.034
Secondary with SLC		1					1			
Tertiary	-0.116	0.89	0.000	0.854	0.929	-0.152	0.86	0.000	0.796	0.926
**Marital status**										
Single	0.059	1.06	0.002	1.022	1.101	0.041	1.04	0.234	0.974	1.116
Married		1					1			
Divorced	-0.008	0.99	0.846	0.913	1.077	0.237	1.27	0.000	1.118	1.436
Widowed	-0.053	0.95	0.817	0.605	1.487	-0.279	0.76	0.425	0.381	1.502
**Constant**	-3.194	0.041	0.000			-4.088	0.017	0.000		
N	88,041					46,127				

Model 1 all mothers, Model 2 – only mothers with a birth order of 2 or more

Model quality Negelkerke R2 is 0.205 for Model 1 and 0.547 for Model 2. The percentage of successfully classified cases is 80.8% for Model 1 and 88.1% for Model 2

Source: [7], own calculations

The odds of a CS decreased with the birth order: for second-time mothers the odds were 11% lower than for first-time mothers (OR = 0.89, 95% CI 0.86–0.93, *p*<0.001) and 25% lower for mothers of higher order births (OR = 0.75, 95% CI 0.71–0.79, *p*<0.001). Women who gave birth to multiple children had 6-times higher odds of a CS (OR = 6.08, 95% CI 5.13–7.21, *p*<0.001) than women with singleton pregnancies. Slightly higher odds of a CS birth were detected for single women (OR = 1.06, 95% CI 1.02–1.10, *p*<0.01) than for married women. No significant difference was observed with respect to the other categories. In terms of the level of education attained, lower odds of giving birth via CS were detected for women with a tertiary education (OR = 0.89, 95% CI 0.85–0.93, *p*<0.001) compared to women with secondary education with SLC.

In terms of health characteristics, the breech position comprises a decisive indication for a CS birth; the odds of a CS birth were more than 30 times higher than for women whose child was in a different position (OR = 31.06, 95% CI 28.14–34.29, *p*<0,001). Women who gave birth pre-term also had higher odds of giving birth via CS, especially those who had very pre-term births (OR = 2.94, 95% CI 2.33–3.71, *p*<0.001). Further, women who most likely became pregnant following embryo transfer had significantly higher odds of a cesarean delivery, even after adjusting for the mother’s age and the birth order and frequency; the odds of giving birth via CS were 1.8-times higher than for women who did not undergo ART (OR = 1.83, 95% CI 1.69–1.99, *p*<0.001). Mothers who suffered from diabetes prior to pregnancy were more than twice as likely to give birth via CS than women who did not have the condition (OR = 2.14, 95% CI 1.76–2.60, *p*<0.001), while those with gestational diabetes had only 1.2-times higher odds (OR = 1.23, 95% CI 1.16–1.31, *p*<0.001). Furthermore, mothers who suffered from hypertension had twice the odds of a CS birth than those without such complications (OR = 2.01, 95% CI 1.86–2.21, *p*<0.001).

### Personal history of cesarean section

Model 2 included only those women who had already given birth (57,960 mothers, i.e. 51.9%), which allowed for the addition of the very significant variable of whether the woman had given birth via cesarean section in the past (Table 4 – Model 2). It was revealed that a previous CS birth comprises an absolutely crucial explanatory variable for a subsequent cesarean delivery. Second and higher-order mothers with previous experience of CS had 32-times higher odds of giving birth via CS than those who had previously given birth vaginally (OR = 32.96, 95% CI 30.95–35.11, *p*<0.001). Either no change was observed with respect to the association of the other monitored variables (diabetes, complications in pregnancy and childbirth, education) or the odds even increased (gestational age, multiple pregnancy and ART use). The odds of CS for women who gave birth very pre-term was 3.5-times higher (OR = 3.56, 95% CI 2.32–5.45, *p*<0,001) than for those who gave birth within term, and the odds of CS for women with a multiple pregnancy was almost 9-times higher (OR = 8.94, 95% CI 6.93–11.54, *p*<0,001) than for those who had a singleton pregnancy.

## Discussion

### Key findings

In accordance with the Robson classification of CS [28], which is accepted as the global standard for the monitoring of the CS indication spectrum [29], a previous CS birth and the breech presentation were confirmed as the highest risk factors for CS birth in Czechia (31-times higher odds of a CS birth for a breech position and 35-times higher odds of a CS birth for a breech position for multiparous women; 32-times higher odds of a CS birth following a previous CS birth) followed by multiple pregnancies (6-times higher odds and 9-times higher odds for multiparous women) and ART use (2-times higher odds). Our analysis also confirmed the importance of the other health and socio-demographic factors examined, i.e. they evinced statistical significance after adjustment for all the other covariates: gestational age, diabetes, complications in pregnancy and childbirth, the mother’s age, marital status and education. The differences in the risk of a CS birth according to marital status and education were statistically significant only for certain categories. A slightly higher risk of CS (1.6-times higher odds) was observed for single compared to married women, and a lower risk of CS (0.89-times lower odds) was observed for tertiary-educated women than for those with a secondary education. Our results confirmed the age factor as an independent risk with concern to a CS birth. With respect to the explanation for the increase in the CS rate in Czechia since the 1990s, both clinical (higher maternal ages at birth, an increase in ART use, multiple pregnancies) and non-clinical factors (health provider practices and guidelines, legislation) played noticeable roles.

### Limitations

Despite the use of a comprehensive dataset, the study has a number of limitations. The design does not allow for the causal interpretation of the associations studied. The covariates in the models were restricted to those available in the register. Information on education and marital status is not provided for all the women in the dataset; hence, for this part of the analysis, it was necessary to reduce the dataset by 21%, although no differences were observed between the two groups in terms of the structure of the mothers by age and CS births. Moreover, information on the use of ART methods was estimated based on information on ART cycles performed in Czechia only; foreign women and women who underwent ART abroad were thus classified as non-ART. Given that Czechia is more likely to be a destination country for cross-border reproductive care, we did not anticipate any bias in the results from this point of view. As for the explanatory factors, since we had no information on the maternal pre-pregnancy weight and height, we were unable to adjust for the body mass index.

### Interpretation

Our results are consistent with literature in terms of reporting significant associations between the studied risk factors and a CS birth. It is reasonable to conclude that these factors have, to various extents, been behind the growth in the CS rate in Czechia since the 1990s. It is important to prevent the further growth in the CS rate and to determine the optimal percentage of CS, especially concerning the elective cesarean delivery of planned primarily indicated CS. It is clear that the underuse of CS results in hypoxic neonatal injury, stillbirth, uterine rupture and obstetrics fistulas [30], while the overuse of CS is associated with the increased risk of anesthesiologic and cardiovascular complications, infection complications and hysterectomy [31], as well as with adverse perinatal outcomes [32].

The incidence of serious complications is so rare due to advances in health care that many obstetricians lack the relevant experience. Nevertheless, the data clearly indicates a higher risk of morbidity and mortality as a result of a CS than a spontaneous delivery, even with respect to VBAC (vaginal birth after cesarean) [33]. However, in Czechia many patients and some obstetricians appear to believe that the opposite is the case, as reflected by the fact that a previous CS was found to be a key risk factor for a subsequent CS. The fact that 71.2% of Czech women with a history of CS give birth again via CS serves to confirm the low chance of a VBAC in such cases. This is in line with another study that documented that a high percentage of births via CS are followed by a subsequent birth via the same method without the option of TOLAC (the trial of labor after cesarean) [34]. Increased maternal age [35] also contributes to the indication of ERCS (elective repeat CS). Enforcing this practice in Czechia may also have contributed to the increase in the CS rate. The increase in women giving birth via CS in their first pregnancy results in an ongoing increase in the repeat CS birth rate [36]. If the CS rate increases for first-time mothers, it can be expected that this will generate a higher proportion of repeat CS. Accordingly, it can be assumed that a change in practice has the potential to reduce the CS rate in Czechia [37].

A further reason for the increase in CS births concerns the move away from spontaneous delivery when the fetus is in the breech presentation. This trend, initiated by the Term Breech Trial Collaborative Group study [38], has gradually led to a decline in the experience of such births and, thus, to a further increase in the use of CS. This approach has begun to be applied consistently in Czechia and is frequently referred to in medical study materials. However, spontaneous delivery when the fetus is in the breech presentation remains inadvisable, especially in the case of pre-term births [39].

The literature shows that a number of maternal health risks are age-related and that the risk of a cesarean birth increases with the maternal age [23, 40, 41]. For example, older mothers are associated with higher risks of the incidence of diabetes mellitus [42], pre-term births [24, 43], lower child birth weights [20, 21, 44, 45] and pre-term births associated with diabetes mellitus [46, 47]. Mothers over 30 years of age also face the increased risk of child health complications, spend longer times in hospital following the birth and face a higher risk of more frequent and longer hospital stays in the first two years of the child’s life [48]. The application of logistic regression confirmed that both pregnancy health complications (preterm-birth, diabetes, hypertension) and the mother´s age comprise independent risk factors for a CS birth. The Czech results confirmed that mothers who gave birth very pre-term (28–31 weeks) had 3-times higher odds of a CS than women who had an in-term birth [49]. Mothers who suffered from diabetes before pregnancy had more than two-times higher odds of giving birth via CS than women who did not suffer from this condition, while mothers with gestational diabetes had 1.23-times higher odds; these results correspond to those of other published studies [50]. As expected, mothers who suffer from hypertension gave birth via CS twice as often as did those with no such complications [51].

Furthermore, our results confirmed the age factor as an independent risk for CS birth. Similar results were reported in a British study [52], the sample population of which comprised 76,158 singleton pregnancies with a live fetus at 11 + 0 to 13 + 6 weeks. After adjusting for potential maternal and pregnancy confounding variables, advanced maternal age (defined as ≥ 40 years) was associated with an increased risk of cesarean section (OR, 1.95 (95% CI, 1.77–2.14); *P* < 0.001). A recent Danish study [12] showed that nulliparous women aged 35–39 years had twice the risk of a CS (adjusted OR, 2.18 (95% CI, 2.11–2.26); *P* < 0.001).

Thus, one of today’s most important population trends – fertility postponement – also comprises one of the significant independent factors associated with the risk of a CS birth. According to Timofeev et al. [22], the ideal age of mothers at birth is 25–29 years, at which time the risk of complications in pregnancy and the neonatal period is lowest. The increased risk of an adverse pregnancy is evident as early as between 30 and 34 years and continues to increase with age [20]. The question thus concerns the age that marks the limit in terms of the increased health risks associated with the mother’s age. The association becomes significant from the age of 40 onwards [52], sometimes even after the age of 35 [12]. In any case, the risks associated with age are of a progressive character [20, 41].

The highest fertility rate in Czechia in 2018 was attained by women aged 30, in contrast to the early 1990s when maximum fertility was attained at the age of 22 [14]. The shift in fertility to older women in Czechia is further illustrated via a comparison of the share of fertility achieved by the age of 30. In 1989, the proportion stood at 86.6%, whereas by 2018 the share had dropped to 48.6% [17]. Thus, the trend toward delayed childbearing is apparent in Czechia as a result of the second demographic transition [53, 54], which indicates that reverse changes in fertility trends are highly unlikely. Nevertheless, fertility postponement can be decelerated or halted by the introduction of effective measures that act to remove barriers to starting a family [14]. To sum up, the strength of the association between advanced maternal age and CS and the fact that the trend in the share of CS births in Czechia has copied the trend in the mean age of mothers at childbirth (Fig. 1) support the hypothesis of a causal relationship between the maternal age and CS. However, as other factors come into play, further research is required so as to assess whether the recent slight decline in the CS rate is not merely a temporal trend.

A further risk factor that is closely connected with fertility postponement concerns the use of ART. Our results confirmed that mothers who most likely became pregnant following embryo transfer also had 1.83 higher odds of a cesarean delivery, even when controlling for the age, order and frequency of birth. According to the meta-analysis of the Medline, EMBASE and CINAHL databases [55], IVF/ICSI pregnancies are associated with a 1.90-fold increase in the odds of a CS (95% CI 1.76–2.06) compared to spontaneous conceptions. Since the late 1990s, Czechia has registered a significant increase in the use of ART and it has become a country with a relatively high proportion of ART live births [18, 19]. Accordingly, the increased use of ART in Czechia may have contributed to the explanation of the increase in the CS rate.

It is noteworthy that, despite the decline in marriage, marital status continues to comprise a relevant variable. In Czechia a slightly higher risk of CS (OR 1.06) was observed for single compared to married women despite the control of variables such as the age of the mother and the birth order. The higher risk of giving birth via CS for single women may be due to the fact that marital status is related to the health status, i.e. married persons have a higher level of self-esteem than do single people [56].

With regard to the level of the woman’s education, no significant differences were detected in terms of the risk of a CS between women with a basic but incomplete education, secondary without the SLC (school leaving certificate) and secondary with the SLC. The controlling of the age and other variables revealed lower odds of a CS birth (OR 0.89) for tertiary-educated women than those with the SLC. The higher odds of CS for women with lower levels of education could be explained by their working in riskier professions, a higher incidence of smoking or obesity or generally poorer living conditions [57]. Conversely, tertiary-educated women are, in general, more open to practicing a healthy life style and receptive to the promotion of the benefits of natural childbirth in contrast to the numerous risks of CS for the subsequent health of both mothers and their children [58]. Thus, the introduction of health education as a component of the antenatal care process as a form of non-clinical intervention should be considered aimed at reducing the unnecessary use of CS [59].

The trend toward an increase in CS in Czechia can also be understood from the legislation perspective, in particular with concern to the introduction of the new Civil Code in 2014, which replaced clearly-defined compensation levels for personal injury with the decision on the amount thereof being decided solely by the courts. The courts continue to maintain the misconception that CS is the best form of intervention in terms of assuring the health of the child and mother. A similar situation has been reported by Longo with respect to Italy [5, 60, 61].

The share of CS births in Czechia (23.6%) exceeds WHO recommendations of 2015 on the optimal proportion of CS births (10–15%). Based on our results, we doubt whether the WHO recommendations reflect the increasingly older ages of mothers, especially first-time mothers and the high degree of institutionalization of deliveries in developed countries. Trusting the delivery to physicians is usually accompanied by a significantly higher degree of monitoring, with the associated risks of false-positive indications of hypoxia, a higher rate of medication use, and the loss of faith in normal childbirth [62].

Some women prefer a CS since they consider it to be safer for both themselves and the baby, an opinion that runs contrary to current scientific knowledge. A history of CS is associated with a higher risk of uterus rupture, placenta accreta, ectopic pregnancy, stillbirth, pre-term birth, and bleeding and the need for a blood transfusion, injury during surgery and hysterectomy in subsequent pregnancies. A higher birth order CS also increases the risk of maternal mortality and morbidity compared to a vaginal delivery [63].

CS may also lead to enhanced health risks for the baby – altered immune development, the increased likelihood of allergies, atopy, asthma, a reduction in intestinal microbiome diversity [64] and late childhood obesity [65]. The risk is higher for planned CS. Few studies have been conducted to date on the influence of CS on the cognitive and educational outcomes of CS-born children [63].

Thus, it is important that all the indications concerning birth via CS are carefully considered and that this method is not overused. Czechia makes no effort to contribute to efforts to reduce the percentage of cesarean sections; on the contrary, the reimbursement of costs by health insurance companies is higher for a cesarean section than for a spontaneous birth. One of the measures that might significantly prevent the expansion of CS use concerns a recommendation from the relevant professional authorities to strictly refuse cesarean sections on request [66]. Although this recommendation has been mentioned frequently in various professional forums in Czechia [67], efforts persist internationally to enforce dubious indications for a CS birth such as the protection of the pelvic floor [68], which also enjoys some support in Czechia. Nevertheless, in Czechia, CS on request is not legally permitted. Furthermore, the implementation of clinical practice guidelines combined with a mandatory second opinion for a CS indication is also relevant to the reduced risk of CS in Czechia [66].

In conclusion, despite the international concern surrounding the increasing CS rate, the Czech CS rate decreased from 26.1% in 2015 to 23.6% in 2018. Interestingly, this has not been attributed to any particular Czech health strategy aimed at reducing the CS rate. Although it has been perceived as a significant success for the field of Czech obstetrics, further research is needed in order to assess whether this is not merely a temporal trend.

### Meaning of the study: possible mechanisms and implications for clinicians and policymakers

Delayed childbearing appears to be associated with the increasing use of CS in parallel with the expansion of defensive obstetrics that imply a high risk of CS in cases of a breech presentation and following a previous CS. In addition, the increased use of CS also reflects social demand, an increasing trend toward the prosecution of obstetricians in the event of childbirth complications and the erroneous lay perception of CS as the safest and least painful childbirth method. On the other hand, clinical practice based on the official refusal of CS on request could well prevent the overuse of CS. As regards obstetric practice, measures to encourage TOLAC, albeit with a careful eligibility assessment, may also help to reduce CS. As regards non-clinical interventions targeted at women, the support of training programs and health education on the indications and contra-indications of CS may also serve to improve the CS rate.

## Conclusion

The aim of the study was to contribute to the explanation of recent trends in the CS rate in Czechia based on the examination of the association between a CS birth and selected health factors and sociodemographic characteristics. Our analysis confirmed that the mother’s age comprises an independent risk factor for a CS birth in addition to pregnancy health complications and other, sociodemographic, characteristics. Accordingly, delayed childbearing appears to be associated with the increase in the CS rate in Czechia. However, the recent slight decline in the CS rate may be related to the completion of the fertility postponement process in Czechia. Nevertheless, since other factors come into play, further research is required in order to assess whether the recent slight decline in the CS rate is not merely a temporal trend.

## Data Availability

The data that supports the findings of this study is available from The Institute of Health Information and Statistics of the Czech Republic (IHIS CR); however, restrictions apply to the availability of the data, which was used under license for the current study; hence the data is not publicly available. The data is, however, available from the authors upon reasonable request and with the permission of the IHIS CR.

## Abbreviations

CS: Cesarean section
NRMC: National Registry of Mothers at Childbirth
IHIS CR: Institute of Health Information and Statistics of the Czech Republic
ART: Assisted reproductive technologies
NRAR: National Register of Assisted Reproduction
OR: Odds ratio
VBAC: Vaginal birth after cesarean
TOLAC: Trial of labor after cesarean
TFR: Total fertility rate
IVF: In vitro fertilization
ICSI: Intracytoplasmic sperm injections
SLC: School leaving certificate
